# A Survey on the Use of Haptic Feedback for Brain-Computer Interfaces and Neurofeedback

**DOI:** 10.3389/fnins.2020.00528

**Published:** 2020-06-23

**Authors:** Mathis Fleury, Giulia Lioi, Christian Barillot, Anatole Lécuyer

**Affiliations:** University of Rennes 1, INRIA, EMPENN & HYBRID, Rennes, France

**Keywords:** neurofeedback, BCI, haptic feedback, EEG, fMRI, multisensory, BMI, touch

## Abstract

Neurofeedback (NF) and brain-computer interface (BCI) applications rely on the registration and real-time feedback of individual patterns of brain activity with the aim of achieving self-regulation of specific neural substrates or control of external devices. These approaches have historically employed visual stimuli. However, in some cases vision is unsuitable or inadequately engaging. Other sensory modalities, such as auditory or haptic feedback have been explored, and multisensory stimulation is expected to improve the quality of the interaction loop. Moreover, for motor imagery tasks, closing the sensorimotor loop through haptic feedback may be relevant for motor rehabilitation applications, as it can promote plasticity mechanisms. This survey reviews the various haptic technologies and describes their application to BCIs and NF. We identify major trends in the use of haptic interfaces for BCI and NF systems and discuss crucial aspects that could motivate further studies.

## 1. Introduction

Over the past decade, advances in brain science and computer technology have led to a growth in the development of neurofeedback (NF) and brain-computer interface (BCI) applications. Recent technological advances, such as machine learning analyses, wireless applications, and real-time neural recordings, have increased interest in NF and BCI approaches, especially EEG-based NF/BCIs. One of the cornerstones of NF and BCIs is the feedback given to the subject, which the subject uses to learn and improve their mental strategy. Traditionally, BCI/NF applications have mostly employed visual feedback, but its use may be questionable in some cases. For example, visual feedback is not always suitable for individuals with an impaired visual system or during a mental motor imagery task, which requires great abstraction from the subject. In such situations, tactile feedback would seem more appropriate and more intuitive than visual feedback (Cincotti et al., 2007b). However, haptic feedback is more often used in conjunction with visual feedback to provide enriched information to the user.

Visual feedback has been shown to be the type of sensory input that produces the best learning processes (Hinterberger et al., 2004). However, up until now, other feedback modalities have been explored less, even though specific circumstances may require differential feedback due to the particular pathology or requirements of the rehabilitation process, e.g., for locked-in patients (Sollfrank et al., 2016). Moreover, it has been suggested that providing haptic feedback could improve the subject's sense of agency, a technology acceptance-related factor, in motor imagery (MI) BCIs (Thurlings et al., 2012). Preliminary studies have shown that BCI performance is not affected by the specific type of feedback (Brouwer and van Erp, 2010), whether visual, auditory, or haptic. Nevertheless, a combination of multiple types of feedback, referred to as multisensory feedback, is expected to provide enriched information (Gürkök and Nijholt, 2012). However, to be efficient, feedback should not be too complex and should be provided in manageable pieces (Lotte et al., 2013).

Haptic feedback is still scarcely used in the BCI/NF community, although the haptic sense is the only one that allows us to interact with the world around us and, at the same time, perceive these interactions (Minogue and Jones, 2006). However, applications related to haptic-based BCIs are myriad, such as in rehabilitation and entertainment. For example, the majority of the clinical papers referenced in this survey focus on stroke patients, because haptic-based BCI/NF seems to be a promising approach to rehabilitation, as such non-invasive techniques may contribute to closing the loop between brain and effect (Gomez-Rodriguez et al., 2011). Haptic-based BCIs could also be used as communication applications to enable patients to perform daily activities independently, e.g., using a wheelchair-driving system (Kaufmann et al., 2013b; Herweg et al., 2016). Given that haptic feedback has evolved over the past decades and haptic displays are becoming increasingly sophisticated, haptic-based BCIs have become unobtrusive and thus more effective and more acceptable to users. In this paper, the term “haptic feedback” encompasses two different types of feedback: tactile and kinesthetic (Figure 4). Tactile feedback refers to the sense of touch, which allows one to recognize texture, pressure, and vibrational stimuli; kinesthetic feedback includes proprioception, which allows one to perceive forces/torques in contact with the body as well as to know the body's position in space, even with eyes closed (Roll et al., 1988).

Haptic interfaces also have different purposes in BCI and NF applications. Historically, NF is used to develop internal control, while BCIs are primarily intended to instruct the control of external objects (an orthosis, a computer, etc.); also, by definition NF is biofeedback from brain areas (Sitaram et al., 2017) with the purpose of self-modulation of brain activity, i.e., for personal control rather than redirection of an object. Following the definitions of NF and BCI, this survey will distinguish the concepts of NF and BCI on the basis of the rationale of their implementation. For example, when a stroke patient uses an exoskeleton for feedback, the goal is not to control that skeleton for the sake of controlling it, but rather to work the perilesional areas in order to activate the plasticity systems. In this case, as the purpose is to enhance neuronal activity, the term NF is appropriate. However, if the paradigm is to control the orthosis, then we will speak of BCI.

In their recent paper, Van Erp and Brouwer (2014) provide an extensive state-of-the-art touch-based BCI. Our survey aims to complement their work with an extension to all haptic modalities/cues and to both BCI and NF applications. Our objective is to better understand the current possibilities of haptic feedback and further improve the design of future studies.

The remainder of this paper is organized as follows. First, we provide an overview of existing haptic technology in section 2. Second, in section 3 we survey recent studies on exploiting haptic feedback in BCI/NF applications, showing the experimental and technical challenges. These works are then discussed in section 4, where we also propose guidelines on the use of haptic technology and identify some remaining challenges. Finally, a conclusion is given section 5.

## 2. An Introduction to Haptic Interfaces

The study of haptic interfaces is an expanding research area focusing on human touch and interaction with the environment through touch. Its integration within BCI experiments is rather recent (since 2007) and was pioneered by Chatterjee et al. (2007) and Cincotti et al. (2007b). The term haptic can be defined as “sensory and/or motor activity of the skin, muscles, joints, and tendons” (ISO, 2011 244: 1). Information delivered through a haptic device is very different from that provided by a visual display. The design of haptic feedback depends on in-depth knowledge of the human haptic sense, either the tactile sense or the kinesthetic sense.

### 2.1. Haptic Perception

**Figure 1:**
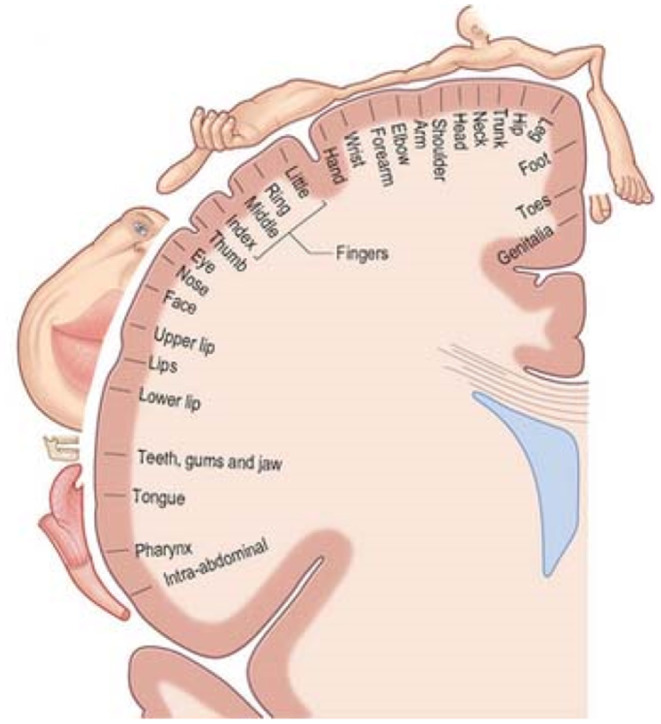
Functional brain areas in the motor cortex (Faller et al., 2012).

The purpose of feedback in a standard BCI/NF protocol is to give cues for a specific type of brain activity in order to have a beneficial impact on the learning of a BCI/NF task (Jeunet et al., 2015). Thus, the effect of the feedback is dependent not only on its content but also on the way it is presented to the subject (Pillette, 2019). For this reason, gaining knowledge of the human haptic sense is a fundamental step in the development of a haptic interface for BCI/NF systems. Haptic interfaces have possible interactions with many parts of the body, which implies that the sense of touch has the potential to become a very useful tool for digital interaction. The human skin is capable of detecting mechanical stimulation, heat, and pain (Aoyagi et al., 2006). When a haptic event occurs, a sequence of voltage pulses is generated and transmitted by the nerves directly to the brain, where the information is processed. For example, picking up an object and sensing its properties (shape, texture, weight, etc.) requires integrating information from the tactile and kinesthetic senses. The primary motor cortex is the physiological location where haptic information is processed. A visualization of a schematic coronal cut showing the distribution of various parts of the body in the primary motor cortex (Figure 1) demonstrates that a considerable proportion is accounted for by the hands and the fingers.

The *tactile sense* is associated with receptors distributed under the surface of the skin. This sense is commonly called the “sense of touch,” as tactile receptors (high-frequency sensors) discriminate very fine surface properties, such as small shapes and fine textures and have a particularly high density under the palm and the fingers (Olausson et al., 2000). On the hands, four types of physiological receptors can be found, as described in Li et al. (2017): “our fingertips can sense a wide range of tactile stimuli, such as temperature, pressure, pain, or vibration.”

The *kinesthetic sense*, or proprioception, is associated with receptors in muscles, tendons, and joints and provides information about the movement, position, and torque of limbs (Antona and Stephanidis, 2015). The term “proprioception” is often used for properties relating to the whole body, whereas “kinesthetic” tends to refer to the perception of properties of limbs; however, this distinction will be neglected in the present survey.

### 2.2. Haptic Interfaces and Actuator Technologies

This section presents the wide spectrum of existing haptic technologies. Haptic feedback can take different forms, but two main categories can be distinguished: tactile feedback and force feedback. Before describing them in more detail, several important properties of haptic interfaces will be provided.

#### 2.2.1. General Properties of Haptic Interfaces

##### 2.2.1.1. Grounded vs. wearable interfaces

**Figure 2:**
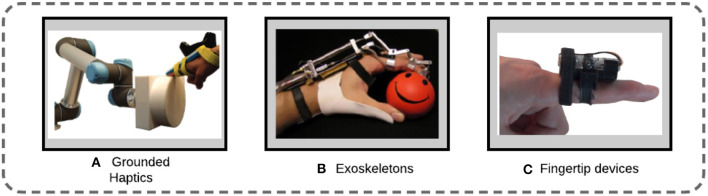
Wearability in haptic devices: from grounded haptic interfaces to more wearable and portable designs. **(A)** ENTROPiA, a cylindrical spinning prop attached to a robot to provide haptic virtual texture (Mercado et al., 2019); **(B)** a hand exoskeleton for natural pitching (Lucas et al., 2004); **(C)** a cutaneous display providing normal and shear stimuli (Pacchierotti et al., 2016).

This categorization is based on whether the haptic interfaces are mobile or anchored to the environment. The design of haptic interfaces recently started to take into account portability as a crucial parameter (Pacchierotti et al., 2017). Furthermore, wearable devices should not limit the user's motion and should enable the stimulation of grasping-related sensations, whereas grounded devices restrain the user's motion and have the ability to stop and block the user. *Ground-based interfaces* are haptic interfaces anchored in the environment. They can generally be classified as link type, magnetic-levitation type, or tension-based type (Kim et al., 2000). The PHANToM, a force-feedback pen with six degrees of freedom (DOF) that provides a force-reflecting interface between a human user and a computer, is an example of a performing link-type haptic interface (Massie and Salisbury, 1994). *Wearable haptic interfaces* are attached to the body of the user. Wearable devices are not limited to a constrained workspace, so they allow users to move freely and perceive haptic feedback in a much larger range. On the other hand, wearability introduces power limitations. Devices must be built with miniature technology, and actuation is limited by weight and power consumption. Pacchierotti et al. (2017) provide a list of guidelines for the design of wearable tactile devices that considers multiple factors, such as the form factor, weight, impairment, and comfort (Figure 2).

##### 2.2.1.2. Active vs. passive touch

Haptic feedback can be divided into two categories: active and passive. Usually active touch refers to the act of touching, while passive touch refers to being touched (Gibson, 1962). For active touch the sensation occurs in the perceiver, and for passive touch it arises in an external device. Hence, passive haptics refer to the haptic properties of physical objects, such as a keyboard or a cup of coffee, and active haptics refer to the haptic properties that are actively generated by the device based on haptic actuators and software. In the haptic field most interfaces are active, but this is not the case for haptic-based BCI/NF systems. Indeed, haptic-based BCI/NF interfaces use calculated feedback from the brain activity and not feedback from the sense of touch. Passive touch is associated with haptic feedback that is not calculated according to the user. For example, a standard vibrotactile alert from a mobile phone can be considered passive feedback.

##### 2.2.1.3. Direct, intermittent, and indirect contact interfaces

**Figure 3:**
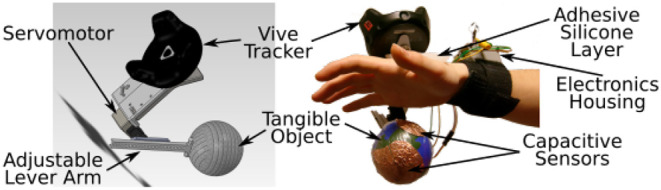
Conceptual schematic of an intermittent contact interface: a tangible object comes into contact with the hand when the finger grabs a virtual ball (de Tinguy et al., 2020).

In the design of a haptic interface, the nature of the contact between the user and the interface can be of three types. *Direct contact interfaces* are attached haptic interfaces such that the user is always in contact with the device. With *intermittent contact interfaces*, user contact with the device is limited and provided only when required. For example, Frisoli et al. (2008) developed a grounded fingertip haptic interface whereby a plate comes into contact with the user whenever their finger touches a virtual surface (Figure 3). *Indirect/mid-air interfaces* produce haptic feedback without having any contact with the user and are therefore not constrained by requirements of the user to wear gloves or hold a device (Bermejo and Hui, 2017). UltraHaptics (Carter et al., 2013), a grounded ultrasonic device, is an example of a mid-air device that provides multi-point haptic feedback to the user's skin. A state-of-the-art review of mid-air devices can be found in Bermejo and Hui (2017).

#### 2.2.2. Tactile Interfaces

Tactile feedback stimulates the surface of the skin through direct contact. Tactile interfaces can be classified according to the sensations they provide: vibration, contact, pressure, temperature, curvature, texture, softness/hardness, and friction (Klatzky and Lederman, 2003). Generally, tactile devices must be lightweight and small, and if the tactile display is to be worn by mobile users, its power consumption must be minimized (Jones and Sarter, 2008). This review will focus on vibration, contact, and pressure interfaces, as these are the most common tactile interfaces in the BCI/NF field. Only feedback related to vibration, contact, pressure, temperature, and electrotactile stimuli will be described in this section; these are the most commonly used types of feedback in the BCI/NF field today.

##### 2.2.2.1. Vibratory feedback

Vibrotactile feedback is generated by mechanical vibration normal or transverse to the skin surface. Mechanical vibration conveys tactile information modulating vibration frequency, amplitude, duration, timber, or spatial location. Vibrotactile feedback uses the same principle as audio headphones, i.e., it converts electrical signals to sound waves. The quality of vibrotactile stimulus perception is influenced by the frequency of the vibration (~50–300 Hz, which corresponds to the bandwidth of the human tactile sense), the body position, and the underlying tissues. The use of oscillating pressure (sinusoidal or square wave and amplitude modulations) also adds new DOF to the design of vibrotactile stimuli, such as waveform and amplitude modulations at different modulation frequencies of the carrier frequency (Klatzky and Lederman, 2003). Vibrotactile devices delivering variable pressure on the skin have been employed, for instance, as alternative sensory channels for blind or deaf individuals (Richardson and Symmons, 1995). The sensitivity of vibrotactile stimulation depends on the body position and age of the subject (Cholewiak and Collins, 2003).

##### 2.2.2.2. Contact and pressure feedback

Contact or pressure feedback can be used to simulate encounters with virtual object surfaces. The effects of such encounters can be simulated with pneumatic systems or surface encounter devices that follow and anticipate the operator's movements (Gabardi et al., 2016). For example, Frisoli et al. (2008) proposed a grounded fingertip haptic interface such that a plate comes into contact with the user when their finger touches a virtual surface.

##### 2.2.2.3. Thermal feedback

**Figure 4:**
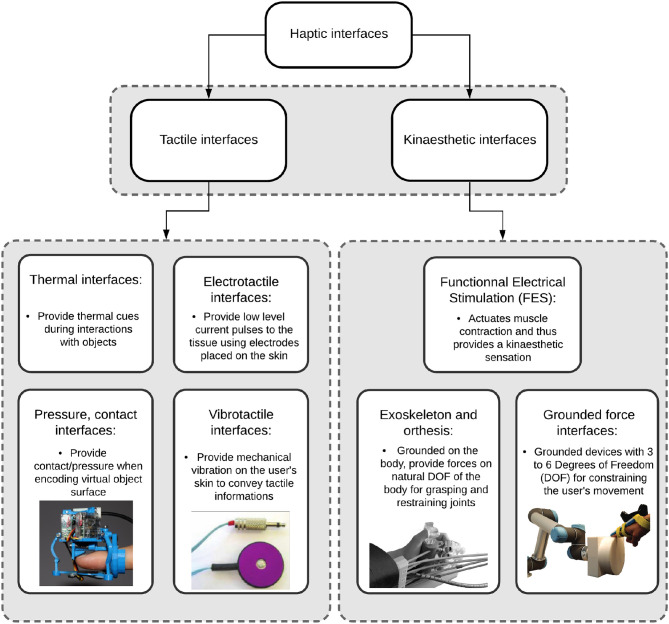
Haptic interface classification. On the left are two representative examples of tactile stimulation interfaces: vibrotactile actuators [C2-tactors (Cincotti et al., 2007a)]; pressure and contact interfaces (Chinello et al., 2012). On the right are two representative examples of kinesthetic stimulation interfaces: Orthosis developed by Ramos-Murguialday et al. (2012); grounded force interface ENTROPiA (Mercado et al., 2019).

Thermal interfaces provide thermal cues to the user that are usually experienced during interactions with objects. Following this principle, Guiatni et al. (2012) created a haptic interface that provides thermal and force feedback for surgical operations (Figure 4). The thermal feedback is matched to the thermophysical properties and temperatures of living organs to aid the surgeon's perception. It has also been proposed to use thermal feedback to make a thermal sensing system for prostheses (Cho et al., 2007). For prosthesis users, thermal stimulation improves their interaction with the surrounding environment and provides them with useful information for everyday activities, such as material discrimination and pain avoidance, as well as psychological comfort. A state-of-the-art survey on thermal displays can be found in Jones and Ho (2008).

##### 2.2.2.4. Electrical feedback

Light electrical stimulation, also known as electrotactile stimulation, can raise the user's awareness and can be used for tactile feedback. Several electrotactile displays have been developed as sensory aids for hearing (Summers et al., 1994) and vision (Kaczmarek and Haase, 2003) and can also be used to create perceptual illusions of surface changes (Wolf and Bäder, 2015). Variations in the intensity and temporal parameters of the stimulation and in the spatial sequence of the electrodes activated can be used to convey information (Jones and Sarter, 2008). However, both the absolute threshold and the subjective magnitude of electrotactile stimulation increase rapidly with changes in the current amplitude (Rollman, 1974). The stimulation current must be controlled carefully to avoid painful sensations in the user. The level of intensity is usually established during a practice session before the recordings. Electrotactile stimulation can also be used for a tongue display unit (Figure 4), which consists of a signal generator that controls the voltage output, a flexible connector cable, and the electrode array. A survey on electrical feedback can be found in Pfeiffer and Rohs (2017).

#### 2.2.3. Kinesthetic Interfaces

In contrast to tactile feedback, force feedback relates to the kinesthetic sense, involving positions, velocities, forces, and constraints sensed through muscles and tendons. Kinesthetic feedback can provide information about limb position and strength applied. Devices that use kinesthetic feedback are usually grounded, since the display of the force or motion is delivered through a tool, such as PHANToM (Massie and Salisbury, 1994) or Omega. However, grasping haptic devices and exoskeletons include wearable devices (e.g., haptic gloves). Haptic clinical devices, such as orthoses or robotic systems have notably been used to guide the movements of paralyzed limbs of patients (López-Larraz et al., 2018).

##### 2.2.3.1. Grounded force feedback

Force-feedback devices usually serve two main purposes: to measure the positions of and contact forces on the user's hand (or other body parts), and to display contact forces and positions to the user. These haptic interfaces are usually composed of rotating joints that connect rigid links (Massie and Salisbury, 1994). Force-feedback devices can be categorized according to the DOF provided by each device, from a simple 1-DOF device to a complex 7-DOF device. Other designs, such as cable systems or stringed haptic interfaces are also grounded force-feedback devices, as they are tension-based systems (Figure 4). Cables are fixed around the corners of a structure, such as a cube. Each cable includes a torque motor, a cable, a tensioning mechanism, and a force sensor. Tension-based haptic interfaces (Sato, 1991) have the advantages of fast reaction speed, simple structure, smooth manipulation, and scalable workspace (Williams, 1998).

##### 2.2.3.2. Exoskeleton devices

An exoskeleton can be used to provide forces on the body with natural degrees of freedom. For example, the orthosis in Figure 4 has to fit the hand naturally without impairing it or interfering with its actions. Heavier exoskeletons can decrease the comfort of the user (Leonardis et al., 2015). The terms orthosis and exoskeleton are used generally to indicate the system effectors, often in an ambivalent way. This review will use the definition from Herr (2009), which stipulates that “generally exoskeleton augments the performance of an able-bodied wearer, whereas orthosis are used to assist a person with a limb pathology and help correct, rehabilitate or support parts of the body.” A review of wearable kinesthetic interfaces can be found in Pacchierotti et al. (2017).

##### 2.2.3.3. Functional electrical stimulation

Functional electrical stimulation (FES), also known as electrical muscle stimulation, is a more intensive type of stimulation [up to 150 V (Pfurtscheller et al., 2003)] than electrotactile stimulation (Li et al., 2017). This kind of electrical stimulation actuates muscle contraction and thus provides a kinesthetic sensation. FES has been used efficiently for motor rehabilitation in stroke patients (Kim et al., 2016; Leeb et al., 2016; Mrachacz-Kersting et al., 2016; Frolov et al., 2017) (Figure 4), showing promising results for motor recovery. A survey on FES can be found in Pfeiffer and Rohs (2017).

## 3. Haptic Feedback in BCI/NF Systems

**Figure 5:**
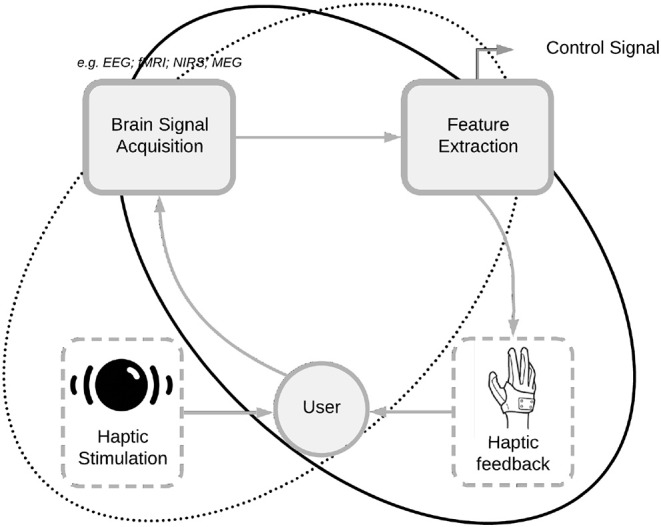
Implementation of haptic feedback in active BCIs (aBCIs) and reactive BCIs (rBCIs). In aBCIs haptic interfaces provide feedback from the user's neural activity, whereas in rBCIs haptic interfaces provide stimulation, and the elicited brain activity is further decoded and transmitted as a command. The aBCI loop is shown as a black solid closed curve and the rBCI loop is represented by the dotted closed curve.

This section describes state-of-the art haptic applications to different BCI and NF paradigms. To date, the MI paradigm is the most used paradigm for haptic feedback, as it offers the possibility of closing the sensorimotor loop: the user imagines a movement and the modulated signal can be employed to control haptic interfaces that in turn give the subject a sensorimotor stimulus. Other paradigms requiring less training, such as P300 and steady-state somato-sensory evoked potential (SSSEP), have also been used in association with haptic interfaces. These haptic sensors are used to stimulate various parts of the body (at different frequencies), and the elicited electroencephalogram (EEG) signals are processed to generate control commands. Haptic displays therefore have different purposes in these two kinds of BCIs: in sensorimotor paradigms they provide haptic feedback from the brain activity of the subject, whereas for P300 and SSSEP haptic interfaces they are used as stimulation, and the evoked brain activity is further decoded to produce a command (Figure 5).

BCIs can be divided into three classes: active, reactive, and passive (Van Erp and Brouwer, 2014). This review considers only BCI applications based on brain patterns and which are actively or reactively generated by the user: active BCIs (aBCIs) and reactive BCIs (rBCIs). An aBCI provides non-muscular communication between the brain and the external environment without external stimuli, for instance in sensorimotor rhythm (SMR) paradigms (Pfurtscheller and Neuper, 1997, 2001; Baker, 2007; Yuan and He, 2014). An rBCI uses external stimuli to provide information to the subject, for example in somato-sensory evoked potential (SSEP) or P300 paradigms. Passive BCIs (pBCIs), which measure the cognitive or emotional state of the subject from brain patterns without any need for specific user activity (George et al., 2012), will be disregarded in the present work.

For an interactive system, the sense of touch is ideal because of its nature. For example, our haptic sense is bidirectional because humans can perceive and actuate via touch (Klatzky and Lederman, 2003). In terms of interface design, this means that touch can be used as both an input and an output tool.

### 3.1. Motor Imagery Paradigms

SMR remains the most popular MI paradigm in haptic-based BCI/NF applications. SMR refers to desynchronization of localized brain rhythms in the upper alpha band (10–12 Hz), usually accompanied by changes in synchronization in the beta band (13–25 Hz) (Pfurtscheller and Neuper, 2001) that occur when performing a real or imagined motor task. This paradigm seems well-adapted to haptic-based BCIs, where tactile and kinesthetic feedback can potentially mimic the natural representation of limb state variables (London et al., 2011). Most SMR-based haptic systems use kinesthetic sensations as feedback from MI performance. The first SMR-based orthosis (hand orthosis, one finger) was designed by Pfurtscheller et al. (2000) for a tetraplegic patient; it was shown that after a period of training (5 months) the patient was able to efficiently control the orthosis with foot or hand MI. Kinesthetic systems differ in their design, which can for instance involve the whole hand or just a few fingers. In most of the studies examined (see Table 1), since the input signal was uni-dimensional, the systems used only one DOF, even if they can deliver more (e.g., the 7-DOF arm orthosis from Gomez-Rodriguez et al., 2011). Different types of movements, such as grasping or opening the hand, can then be transmitted. Grounded systems are typically used for kinesthetic feedback because orthoses are heavy (Buch et al., 2008; Ang et al., 2010, 2014; Ramos-Murguialday et al., 2012; Ono et al., 2014; Darvishi et al., 2017; Chowdhury et al., 2018). However, portability is an important factor for haptic interfaces, which should not limit the motion of the user. Based on this consideration, some studies have investigated portable kinesthetic feedback (Chen et al., 2009; Bundy et al., 2017; Frolov et al., 2017).

**Table 1:** SMR-based haptics.

**Haptic sensation**	**Actuator technology**	**Portability**	**MI task**	**Multimodality**	**Haptic gain**	**C/D**	**Purpose of study**	**NP/NS**	**References**
K	H orthosis (3 F)	G	Grasp H	V (grasping H), PP	N/A	C	Rehabilitation	4/– SP	Chowdhury et al., 2018
K	H orthosis (H flexion)	G	Open H	V (bar)	Sup	D	Research HI	–/10	Darvishi et al., 2017
K	H orthosis (all F)	G	Open/close H	None	N/A	C	Research HD	–/23	Ramos-Murguialday et al., 2012
K	H orthosis (all F)	G	Reach/grasp/bring H	None	N/A	C	Rehabilitation/Clinical	16/16 SP	Ramos-Murguialday et al., 2013
K^*^	H orthosis (4 F)	G	Open/grasp H	V (bar)	N/A	D	Rehabilitation/Research SP/HD	8/– SP	Buch et al., 2008
K^*^	H orthosis (all F)	G	Moving H	None	N/A	C/D	Rehabilitation	4/20 SP	Soekadar et al., 2011
K^*^	H orthosis (all F)	G	Open H	None	N/A	C	Research HD	–/30	Soekadar et al., 2015
K	H orthosis (H flexion)	G	Open F	V (grasp H)	=	D	Rehabilitation/Research FC	12/– SP	Ono et al., 2014
K	H orthosis (all F)	P	Open H	V (clue/color change)	N/A	C	Rehabilitation/Clinical	55/19 SP	Frolov et al., 2017
K	H orthosis (2 F)	P	Open H	V (clue)	N/A	C	Rehabilitation/Research brain location	10/– SP	Bundy et al., 2017
K	H orthosis (2 F)	P	Open/grasp H	V (bar)	N/A	D	Research HD	–/11	Chen et al., 2009
K	H orthosis (2 F)	P	None	None	N/A	D	Rehabilitation	6/– TP	Soekadar et al., 2016
K	H orthosis (1 F)	P	Not specific	V (bar)	N/A	D	Rehabilitation	1/– TP	Pfurtscheller et al., 2000
K	Arm orthosis	G	Flexion/extension forearm	V (arrow)	N/A	D	Rehabilitation/Research HI	2/6 SP	Gomez-Rodriguez et al., 2011
K	Arm orthosis (2 DOF)	G	Arm direction	V (target)	N/A	D	Rehabilitation	54/– SP	Ang et al., 2010
K	H knob	G	Grasp H	V (cue)	N/A	D	Rehabilitation	21/– SP	Ang et al., 2014
T-Vib	Mechanical vibrators (on the biceps)	P	H right/left	V (bar)	N/A	C	Research HD	–/6	Chatterjee et al., 2007
T-Vib	Mechanical vibrators (upper part of trunk)	P	H right/left	V (bar)	=	D	Research/Rehabilitation FC	30/3 spinal cord injuries patients	Cincotti et al., 2007b
T-Vib	Mechanical vibrators (neck)	P	H or foot	V (bar)	=	C	Research FC, HI	–/6	Leeb et al., 2013
T-Vib	Eccentric rotating mass (neck)	P	Tapping with F	None	N/A	D	Research	–/11	Liburkina et al., 2018
T-Vib	Gloves with 5 eccentric rotating mass vibrators per H	P	H right/left	V	N/A	C	Research/Entertainment FC	–/18	Jeunet et al., 2015
E-T	Tongue display unit array	P	H and foot	V (bar)	=	C	Rehabilitation/Research HD & FC	1/10 BP	Wilson et al., 2012
FES	ES of the forearm	P	Open H	None	N/A	D	Rehabilitation	16/– SP	Leeb et al., 2016
FES	ES of the forearm	P	H and foot	None	N/A	D	Rehabilitation	1/– TP	Pfurtscheller et al., 2003

*K, kinesthetic*;

* *, MEG; T-Vib, tactile-vibrotactile; E-T, electrotactile; FES, functional electrical stimulation; H, hand; F, finger(s); DOF, degrees of freedom; ES, electrical stimulation; P, portable; G, grounded; V, visual; PP, physical practice; C, continuous; D, discrete; HI, haptic influence; HD, haptic design; FC, feedback comparison; NP, number of patients; NS, number of subjects; SP, stroke patient; TP, tetraplegic patient; BP, blind patient*.

Haptic feedback can be delivered both continuously (where the feedback is given during execution of the mental task and directly reports the neural activity) and discretely (where the feedback is given after reaching a threshold). For example, Murguialday et al. (2011) proposed a system composed of a mechanical hand orthosis attached to the upper limb that can extend and close all fingers in order to investigate the effect of proprioception on BCI control. They showed that in healthy subjects, SMR-based BCI/NF training with contingent haptic feedback improves BCI performance and motor learning, enhancing SMR desynchronization during MI. These results were also found by Soekadar et al. (2011), who showed that graded haptic feedback outperforms binary feedback, leading to faster BCI learning and more accurate SMR-ERD modulation (where ERD stands for event-related desynchronization).

The use of tactile feedback for SMR-based BCI/NF systems has also been developed, because of its greater portability, comfort, and affordability relative to kinesthetic interfaces. Tactile interfaces have been used to unload the visual channel (Cincotti et al., 2007b; Leeb et al., 2013; Liburkina et al., 2018) for individuals with impaired vision (Wilson et al., 2012) and patients with spinal cord injury (Cincotti et al., 2007b). Chatterjee et al. (2007) demonstrated that users can control a BCI system using only tactile feedback with vibrotactile stimulators placed on the right or left upper arm. They found that vibrotactile feedback helped the subject regulate contralateral imaginary tasks. In a lingual electrotactile study, Wilson et al. (2012) demonstrated that task performance with tactile feedback was comparable to that with visual feedback. In an extended experiment involving 30 healthy participants and three subjects with spinal cord injury, Cincotti et al. (2007b) showed that the vibrotactile channel can function as a valuable feedback modality, especially when the visual channel is loaded by a secondary task.

Even though the first study of haptic feedback in clinical applications was a case report on a tetraplegic patient (Pfurtscheller et al., 2000), a large proportion of these studies focus on stroke rehabilitation (Buch et al., 2008; Ang et al., 2010; Gomez-Rodriguez et al., 2011; Soekadar et al., 2011; Ramos-Murguialday et al., 2013; Ono et al., 2014; Frolov et al., 2017; Chowdhury et al., 2018). Haptic-based MI BCIs are promising for functional rehabilitation of stroke patients, as such training can also be used with patients having no residual movement. The aim of such BCI/NF systems is to stimulate neural plasticity in perilesional brain motor areas and support upper limb functional improvement (Lioi et al., 2018, 2020). Since haptic BCI/NF-based SMR techniques achieve motor imagery with concurrent motor learning via kinesthetic feedback, it is natural to think of rehabilitation of stroke patients even in a chronic condition. In these applications the question of the cortical target is still open. Usually control of the orthosis is modulated by the ipsilesional side of the brain (Pichiorri et al., 2015), contralateral to the affected hand; however, the ability to modulate perilesional activity decreases with increased cortical damage (Buch et al., 2012). For example, Bundy et al. (2017) studied the contralesional motor area for control of a portable exoskeleton, the assumption being that recovery would be optimal on the contralesional side and that functional improvements may be elicited (Ward et al., 2003). In 2008, Buch et al. (2008) demonstrated that chronic stroke patients with upper limb hemiplegia were able to control a magnetoencephalography (MEG) BCI by voluntarily modulating the ipsilesional SMR amplitude while receiving contingent haptic feedback with a hand orthosis. The haptic system used was a grounded mechanical orthosis attached to the plegic hand, with one attachment on each finger except the thumb. The feedback was given in a passive way, with a movement of the orthosis elicited only if the modulation had reached a certain threshold at the end of the trial. Kinesthetic feedback is mostly employed for stroke rehabilitation, in agreement with the finding that rehabilitation of motor functions is more efficient with proprioceptive feedback. Most studies of rehabilitation involve kinesthetic feedback, but FES-based MI has also been performed for patients. In an early study by Pfurtscheller et al. (2003), they reported applying non-invasive techniques to restore grasp functionality in a tetraplegic patient through FES. This same method was used with chronic stroke patients in Leeb et al. (2013). The interested reader can find more information about BCI applications for stroke rehabilitation in the 2018 review article by López-Larraz et al. (2018).

### 3.2. External Stimulation Paradigms

Brain signals can be elicited by using external stimulation. Frequently used paradigms include SSEP and event-related potentials (ERPs). Most BCIs using ERPs can be employed without any prior training and do not exhibit the so-called “BCI illiteracy” problem (where the BCI system fails to correctly detect the mental state of its user). The following paragraph will deal with external paradigms (P300 and SSSEP) and their relationship with the haptic modality. To the best of our knowledge, in contrast to SMR-based BCI/NF systems where haptic technologies are used to provide the feedback, in external stimulation paradigms haptic interfaces are mostly employed as a stimulus.

#### 3.2.1. P300

P300 is a positive deflection of the EEG signal occurring around 300 ms after presentation of a given stimulus (visual, haptic, or auditory). A major strength of the P300 paradigm is its reproducibility and stability as a feature of rBCIs (Donchin et al., 2000). The majority of P300-based BCI studies use the visual channel for stimulation (Table 2): one motivation for using haptics for P300-based BCIs is in fact to reduce the dependence of the gaze in rBCIs. The interest here is not so much to imitate a kinesthetic or tactile sensation but rather to give the haptic stimulation in the most efficient way. Indeed, most haptic-based P300 studies use tactile rather than kinesthetic sensation as stimulation. The first to adopt this paradigm in a haptic-based BCI study were (Aloise et al., 2007), who investigated the influence of a tactile stimulus on classification performance in eight subjects. The tactile stimulus was provided with eight vibrotactile stimulators placed at different positions on the hands and wrists. The authors reported that the tactile stimulus increased the latency of the principal P300 component (a 600 ms peak after the haptic stimulus against 400 ms for the visual stimulus) and that online classification performance was weaker than with a visual stimulus (68 against 93%). Other studies using vibrotactile tactors in P300-based BCIs followed, differing in where the vibrators were located: on the wrist, on the arm, on the palm (Rutkowski and Mori, 2015), on the neck, or even on the head (Mori et al., 2013).

**Table 2:** P300-based haptics.

**Haptic sensation**	**Actuator technology**	**Portability**	**Multimodality**	**Haptic gain**	**Purpose of study**	**NP/NS**	**References**
T-Vib	Mechanical vibrators H or W	P	V	Inf	Research FC	–/18	Aloise et al., 2007
T-Vib	Mechanical vibrators (waist)	P	None	N/A	Research HD	–/10	Brouwer and van Erp, 2010
T-Vib	Mechanical vibrator (left/right W)	P	None	N/A	Rehabilitation	11/– LIS	Lugo et al., 2014
T-Vib	Mechanical vibrators (left/right W)	P	None	Sup in communication speed	Research FC (auditory and MI)	–/10	Qiu et al., 2017
T-Vib	Mechanical vibrators (left/right W & shoulder)	P	None	N/A	Rehabilitation	12/– LIS/CLIS	Guger et al., 2017
T-Vib	Mechanical vibrators (4 on arm)	P	None	Sup	Rehabilitation/Research FC	1/– LIS	Kaufmann et al., 2013b
T-Vib	Mechanical vibrators (torso)	P	V	Bimodal = unimodal	Research	–/10	Thurlings et al., 2012
T-Vib	Mechanical vibrators (3 F)	P	Hex-O-Spell	Inf	Rehabilitation/Research FC	6/5 ALS	Severens et al., 2014
T-Vib	Mechanical vibrators (knees, abdomen, neck)	P	None	N/A	Research HD	–/10 elderly subjects	Herweg et al., 2016
T-Vib	Mechanical vibrators (F)	G	Hex-O-Spell	=	Research HD FC	–/12	van der Waal et al., 2012
T-Vib	Mid-air stimulation	G	None	N/A	Research HD	–/13	Hamada et al., 2014
T-Pressure	solenoids (F: I,M,R)	G	V	N/A	Research HD	–/5	Shimizu et al., 2014
K	H FF	G	None	N/A	Research HD	–/7	Kono et al., 2013

*T-Vib, tactile-vibrotactile; K, kinesthetic; H, hand; W, wrist; F, finger(s); T, thumb; I, index finger; M, middle finger; R, ring finger; FF, force feedback; P, portable; G, grounded; V, visual; FC, feedback comparison; HD, haptic design; MI, motor imagery; NP, number of patients; NS, number of subjects; (C)LIS, (complete) locked-in syndrome; ALS, amyotrophic lateral sclerosis*.

The use of other forms of haptic interfaces in P300-based BCIs is still marginal, and further studies are required to assess if they have the potential to enhance BCI efficiency. Kinesthetic stimulation with force feedback was investigated in Kono et al. (2013), where the kinesthetic sensation was delivered through a joystick to the subject's dominant hand and provided four different movements corresponding to the four main directions. Hamada et al. (2014) tested the first non-contact method of producing tactile sensations for BCIs (mid-air haptics), and in Shimizu et al. (2014) tactile pressure sensation was tested.

The P300 paradigm requires less training and may achieve higher accuracy than the MI paradigm (Allison and Neuper, 2010), as well as having the potential to be used in the control of communication systems for patients with locked-in syndrome (LIS) or completely locked-in syndrome (CLIS). LIS or CLIS are conditions where the patient cannot communicate or does not have control over their motor functions except for vertical eye movements and blinking (Duffy, 2012). BCIs may offer a new communication solution for such patients who have sufficiently intact cognitive abilities (Wolpaw et al., 2002; Karim et al., 2006). It is from this perspective that Guger et al. (2017) compared the two paradigms to assess whether vibrotactile P300 could outperform MI in a communication system for LIS patients. The use of haptic-based P300 for control of an object in the everyday environment has also been studied, in particular in the context of wheelchair control, because visual feedback limits the user's interaction with the external environment (Herweg et al., 2016). Recently, a spelling application involving the use of tactile stimulation on the fingertips was developed by van der Waal et al. (2012), with resulting spelling rates similar to those of visual spellers. Kaufmann et al. (2013a) described an experiment in which they tested healthy users steering a virtual wheelchair in a building. The four navigational directions were associated with different tactor locations on the body. Out of the 15 participants, 11 successfully navigated a route along four waypoints, supporting the view that the haptic P300 paradigm has potential for medical applications.

#### 3.2.2. Steady-State Somatosensory Evoked Potentials

SSSEPs constitute a steady-state component of brain signals evoked by sustained repetitive vibrotactile stimulation within the frequency range of 17–35 Hz (Breitwieser et al., 2012). The idea behind the method is to increase the information transfer rate (ITR) (which is slower with SMR-based paradigms because it requires some time to establish ERD patterns) without loading the eye gaze (Müller-Putz et al., 2006). SSSEPs also represent an alternative to visual-based P300 or steady-state visual evoked potential (SSVEP) systems. Because the stimulation paradigm is based on vibrations, most studies employ tactile interfaces, using a vibrator to deliver the stimulus (Table 3). The first appearance of SSSEP within a haptic-based BCI environment is in the study by Müller-Putz et al. (2006), in which the authors investigated whether SSSEP is as efficient as the BCI paradigm. Tactile stimulation was provided by vibrotactile stimulators placed on both index fingers, and the user had to concentrate on one stimulus (right or left) at a time. Müller-Putz et al. reported that of four healthy subjects, only half attained a classification accuracy of 70%. The placement of the vibrotactile stimulators in SSSEP-based BCIs differs between studies, though in most cases the stimulators are concentrated on the user's hands (fingers, wrist) or feet (Kim and Lee, 2017), as the discrimination of different vibration frequencies is higher when the tactors are placed in these locations. Comparison of different paradigms has also been done; for example, Severens et al. (2013) studied the difference between the performance of SSSEP and that of P300, reporting that P300 outperformed SSSEP and that the combination of the two did not result in better performance than with P300 alone. These results show the limitations of the SSSEP paradigm: the comfort of subjects is low (they have to concentrate on one of two or more tactile stimuli) (Ahn et al., 2016), which is not the case with SSVEP, where eye position primarily determines the target (Müller-Putz et al., 2006). Combining SSSEP with other paradigms could be a more promising approach. Ahn and Jun (2012) combined SSSEP (left and right fingers) with an imagined-movement BCI paradigm. Kim and Lee (2017) designed a wheelchair-driving system that uses three vibrotactile stimulators to control different directions, indicating that this system has the potential to help amyotrophic lateral sclerosis (ALS) patients or other patients with LIS gain independence in their daily activities.

**Table 3:** Haptic-based SSSEP.

**Haptic sensation**	**Actuator technology**	**Portability**	**Multimodality**	**Haptic gain**	**Purpose of study**	**NP/NS**	**References**
T-Vib	Mechanical vibrator (1 F)	P	None	N/A	Research	–/4	Müller-Putz et al., 2006
T-Vib	Mechanical vibrator	P	None	N/A	Research	–/14	Breitwieser et al., 2011
T-Vib	Mechanical vibrator (W)	P	V (cue)	N/A	Research	–/57	Yao et al., 2017
T-Vib	Mechanical vibrator (2 F)	G	None	N/A	Research	–/16	Ahn et al., 2014
T-Vib	Mechanical vibrator (left/right foot)	P	None	N/A	Research/Rehabilitation	–/5	Kim and Lee, 2017

*T-Vib, tactile-vibrotactile; F, finger; W, wrist; P, portable; G, grounded; V, visual; NP, number of patients; NS, number of subjects*.

## 4. Discussion and Perspectives

Haptic-based BCI/NF applications have gained increasing interest in recent years. Researchers using haptics to provide feedback or stimulation have focused on three different paradigms: (1) haptic-based SMR, which mostly employs kinesthetic feedback and is used for stroke rehabilitation; (2) haptic-based P300, where tactile stimuli are generally used to elicit a brain response for the control of an object; and (3) haptic-based SSSEP, in which vibrotactile stimuli are employed. In each of the paradigms discussed in this review, clinical applications have been tested with promising results. Nevertheless, there are limitations and challenges that must be addressed by the haptic-based BCI community. In this section, we discuss some points regarding the design of haptic systems adapted to BCIs, the utility and interest of haptic feedback for BCI and NF applications (compared with other modalities), and limitations of the current solutions.

Most BCI studies involving haptics have used the MI paradigm, often in conjunction with visual feedback. This general trend is mostly explained by the fact that in an MI task, closing the sensorimotor loop has the potential to improve the quality and pertinence of the feedback provided, thus enhancing user engagement and NF performance. On the other hand, for the SSSEP and P300 paradigms, haptic feedback is seen more as an alternative to the visual channel. Concerning the applications of haptic BCI/NF, this review indicates that there is a major tendency toward using these systems for rehabilitation, especially for stroke patients, and that the vast majority of studies used kinesthetic feedback, with the goal of reproducing a real and complex movement. On the other hand, tactile feedback is mainly used with the aim of restoring comfort for patients with LIS or visual impairments, rather than for rehabilitation purposes. The majority of kinesthetic feedback involves the upper limbs, with orthoses placed on either the hand or the arm; this is not necessarily the case for tactile feedback, which can be placed on different parts of the body. The visual modality is also commonly employed in these studies, either to provide a visual clue (i.e., to know if the user has to imagine a right or left movement) or as feedback complementary to the haptic feedback. The visual feedback either takes a classic form (e.g., a bar or thermometer) or is a more realistic kind of proprioceptive feedback representing, for instance, a hand. The gain of the haptic modality with respect to the visual modality in different paradigms still needs to be more accurately assessed, although several studies have paved the way and seem to converge on the finding that haptic feedback is either equivalent to or more effective than visual feedback in certain applications.

Haptic-based P300 mostly uses tactile vibration as a stimulus, and rarely other modalities. Visual stimuli are used together with haptic stimulation in the P300 paradigm mainly to assess the gain of a haptic stimulus. There is still no consensus around this gain, as some studies show an equivalent effect on the classification performance while in others haptic-based paradigms have reduced performance relative to visual ones. The use of a haptic stimulus is often motivated by the fact that haptic stimulation is the only possible communication channel for some patients (e.g., those with LIS or CLIS), for whom use of the visual channel is not always possible. In contrast to haptic-based SMR paradigms, for P300 applications there is a richer literature dealing specifically with the design of the haptic interface.

Applications based on haptic SSSEP are very similar to P300 paradigms, but limited research has been done on the design of such systems. Since P300 and SSSEP share similar objectives, it would be interesting to compare these two paradigms in future studies.

### 4.1. Design of Haptic-Based BCI/NF Systems

The integration of haptics in BCI/NF environments can be complex and entails some challenges at different levels. This is also because haptic-based BCI/NF studies are usually designed by imitating visual feedback protocols, even if the design may be sub-optimal for the haptic modality. In this section, we discuss some issues that should be addressed in the design of haptic BCI/NF protocols adapted to specific applications.

#### 4.1.1. When and How Should the Feedback Be Provided?

The basis of human-computer interaction is the use of feedback, which underlies the interactions occurring between the user and the system (Hewett et al., 1992). A recurring question in the BCI community is the frequency at which the feedback should be provided. The feedback can be given in two different ways: continuously or discretely. It would seem more natural in a BCI environment to give the feedback at the end of a successful trial rather than continuously. By contrast, in an NF paradigm, the feedback is an indicator to the user of their own cerebral activity, so in this case it would seem more appropriate to give haptic feedback in real time. A recent study (Shu et al., 2017) indicates that improvement in an MI task could be achieved if vibrotactile stimulation of the non-dominant or paretic hand of the patient is performed during MI, thus highlighting the importance of defining the feedback delivery modality depending on the desired application.

#### 4.1.2. Artifacts Induced by Haptic Interfaces

In haptic BCI/NF applications, various artifacts can contaminate the signal; these can be generated by the devices controlled with the haptic feedback [e.g., noise generated by actuators based on electric/magnetic neurostimulation or by robotic devices (Leeb et al., 2013; Insausti-Delgado et al., 2017)] or have a physiological origin (e.g., compensatory movements, cranial and neck muscle activity, eye movements, swallowing, etc.). The question of whether the haptic feedback introduces additional artifacts that influence BCI performance is still debated, and the answer depends greatly on which haptic system has been tested. For tactile feedback, some studies have shown no interference with the electric signal (Kauhanen et al., 2006; Chatterjee et al., 2007). For example, Leeb et al. (2013) demonstrated no significant difference during the rest and the stimulation with vibrotactile feedback. However, Hommelsen et al. (2017) showed FES feedback to be a considerable source of false positives when the mu rhythm was used for the detection of efferent commands. We suggest that a thorough study of the influence of haptic feedback, whether tactile or kinesthetic, be conducted to determine what kinds of artifacts are induced by vibratory feedback and feedback with an orthosis.

#### 4.1.3. Features Extraction and Feedback Calculation

According to recent findings of Bashashati et al. (2015), the choice of the classifier for a BCI system depends on the feature extraction method used. We also recommend that the choice of classifier and the choice of feature take into account the specific feedback modality employed; for example, an optimal classifier for haptic feedback may not be efficient for visual feedback. The majority of EEG classification algorithms were developed for vision-based BCI/NF, while neurophysiological responses to tactile stimuli may differ; a research effort to define a methodological framework specific to the analysis of haptic features is therefore needed.

#### 4.1.4. Haptic-Based BCI/NF vs. Haptic Interfaces: A Technological Gap

To date, the BCI community has been using haptic interfaces for sensory feedback or as stimulation systems that are generally simple and sometimes dated. The design of haptic interfaces has progressed hugely in recent years, and it would be interesting to integrate the technological advances into BCI/NF studies. If we consider, for instance, the DOF of haptic devices, at present the majority of studies involving a kinesthetic system are limited to only one DOF, even if the device can provide more. Using more DOF may facilitate motor learning (Scheidt et al., 2000) and should be investigated for rehabilitation of stroke patients. For stroke rehabilitation, tactile or kinesthetic devices already exist but not in a BCI environment; for example, Lin et al. (2016) developed a haptic glove equipped with vibrotactile stimulators that interact with a virtual-reality environment. Other studies have focused more on ergonomics for the user by designing exoskeletons with multiple DOF (Wege and Hommel, 2005; Godfrey et al., 2013). However, wearability is often not a priority, though it must be taken into account to enable the user to optimally perceive and interact with the environment. For example, In et al. (2011) developed wearable hand interfaces and proposed a jointless hand exoskeleton weighing only 80 g. We suggest that portability of haptic feedback should become more central to the study of haptics design in the future.

### 4.2. Haptic vs. Other Modalities

Visual feedback has historically dominated the field of BCI/NF, and only in recent years have other modalities (auditory or haptic) for delivering information been explored (Lukoyanov et al., 2018).

#### 4.2.1. The Gain of the Haptic Modality

The gain of the haptic modality over other modalities can be assessed looking at different parameters, such as BCI/NF performance, comfort of the subject, or adaptation in an everyday environment. For example, haptic feedback could enhance MI (Shu et al., 2017) by bypassing BCI illiteracy. BCI illiteracy represents a big challenge in BCI research (Vidaurre and Blankertz, 2010), and currently available SMR-based BCI/NF systems may have reached the limits of their performance, as ~30% of healthy subjects (Blankertz et al., 2010) and 40% of stroke patients (Ang and Guan, 2015) cannot attain the critical BCI accuracy of 70%. Recent work of Lukoyanov et al. (2018) suggests that after some training, the type of feedback (visual or tactile) does not affect the classification accuracy. It does, however, affect the comfort of subjects, who describe tactile feedback as being more natural. Moreover, there are still few studies that compare different modalities: for SMR paradigms it seems that the visual and haptic modalities are comparable in terms of BCI performance; however, for P300-based studies this is still not clear. The gain of the haptic modality must also be assessed with respect to the decrease in visual workload, since the feedback no longer occupies the visual channel.

In current approaches haptic feedback is delivered in a uni-dimensional way; for example, the task performed by the user is usually binary, such as open/close or open/grasp. For stroke rehabilitation this could be a limitation, since the mental task is often more complex in reality. Future studies should explore the possibility of including more than one task in order to provide more complex training (bearing in mind that this would also increase the training time). We recommend that further research be done on the design of more realistic haptic training.

#### 4.2.2. Multimodality

In our daily environment we encounter many simultaneous and multimodal stimuli. It is therefore of interest to test a multisensory feedback approach in a BCI context. One might hypothesize that multimodal feedback, such as visuohaptic or audiovisual feedback would be more effective than simple unimodal feedback (Sigrist et al., 2013). In a clinical context this might also be interesting to investigate; for example, vision may be compromised in LIS, CLIS, or ALS patients, and additional sensory feedback may provide a good alternative to uni-dimensional feedback. Several studies have tested the impact of multimodal visuo-auditory feedback for BCI-based SMR; overall the multimodal feedback was found to either have a similar effect to unimodal visual feedback (Schreuder et al., 2010) or yield better results in the first session (Sollfrank et al., 2016). In some cases multimodal feedback increased performance in some naive subjects (Gargiulo et al., 2012). For the visuohaptic modality, an investigation of Brouwer and van Erp (2010) showed that visual-tactile feedback has better performance than uni-sensory stimulation. It has also been suggested that the feedback given to the subject could be either equally shared between different channels, replicated on each channel (Cincotti et al., 2007b), or even dynamically distributed between channels. Although the use of visual feedback in addition to haptic feedback is often systematic, it is not always justified. This suggests that further work is needed to shed light on the use of multimodal feedback and to assess the efficacy of visuohaptic feedback compared to unimodal feedback, whether visual or haptic.

## 5. Conclusion

Haptic interfaces are undergoing major technological progress, and the BCI/NF community is looking at the haptic modality with increasing interest. In this review we have summarized and discussed the state-of-the-art research on haptic-based BCI/NF applications. We have outlined different paradigms using haptic interfaces, such as SMR, P300, and SSSEP, as well as methodologies for the design of pertinent haptic applications. We have identified major trends in the use of haptics in BCIs and NF and discussed the limitations of current solutions. To date there is no consensus on the effectiveness of haptic feedback for BCI and NF systems. This review shows that haptic interfaces have the potential to enhance performance and increase the pertinence of the feedback provided, in particular for the SMR paradigm, which is used in the context of motor rehabilitation. Further studies are, however, needed to test the use of innovative haptic technologies for BCI and NF and to assess the utility of the haptic modality, used either alone or in combination with other modalities.

## Author Contributions

MF, GL, CB, and AL contributed the conception and design of the review. MF wrote the first draft of the manuscript, wrote the sections of the manuscript, and organized the database. GL wrote the sections of the manuscript. All authors contributed to the article and approved the submitted version.

## Conflict of Interest

The authors declare that the research was conducted in the absence of any commercial or financial relationships that could be construed as a potential conflict of interest.
